# The Interaction Between Mental Distress and Opioid Maintenance Treatment Impacts Levels of Circulating Cytokines

**DOI:** 10.1111/adb.70147

**Published:** 2026-03-29

**Authors:** Kristin Nygård‐Odeh, Hedda Soløy‐Nilsen, Magnhild Gangsøy‐Kristiansen, Ole‐Lars Brekke, Tom Eirik Mollnes, Michael Berk, Jørgen G. Bramness

**Affiliations:** ^1^ Nordland Hospital Trust Bodø Norway; ^2^ Institute of Clinical Medicine UIT ‐ The Arctic University of Norway Tromsø Norway; ^3^ Research Laboratory Nordland Hospital Trust Bodø Norway; ^4^ Department of Immunology Oslo University Hospital and University of Oslo Oslo Norway; ^5^ Deakin University IMPACT – The Institute for Mental and Physical Health and Clinical Translation, School of Medicine, Barwon Health Geelong Australia; ^6^ Orygen, the National Centre of Excellence in Youth Mental Health, Centre for Youth Mental Health, Florey Institute for Neuroscience and Mental Health and the Department of Psychiatry The University of Melbourne Melbourne Australia; ^7^ Norwegian National Advisory Unit on Concurrent Substance Abuse and Mental Health Disorders Innlandet Hospital Trust Brumunddal Norway; ^8^ Department of Alcohol, Tobacco and Drugs Norwegian Institute of Public Health Oslo Norway; ^9^ Section for Clinical Addiction Research Oslo University Hospital Oslo Norway

**Keywords:** cytokines, mental health, opioid maintenance treatment, psychiatric symptom clusters

## Abstract

Opioid use modulates parts of the immune system, including cytokines, but with disparate results. Furthermore, several studies have demonstrated a positive correlation between circulating proinflammatory cytokines and mental distress of various kinds. The aim of this study was to investigate the relationship between self‐reported mental health symptoms and peripheral circulating cytokines in opioid maintenance treatment patients to see whether the previous disparate results could at least in part be explained by an interaction with mental distress. In a cross‐sectional study, we investigated levels of 27 serum cytokines and chemokines using multiplex technology in 120 patients with chronic hepatitis C virus infection. Self‐reported mental health symptoms were obtained using SCL‐90‐R. Among the nonopioid maintenance treatment patients, we found a positive correlation between self‐reported mental health symptoms and peripheral circulating cytokines. An opposite trend was found for several of the proinflammatory cytokines in the opioid maintenance treatment patients, which was confirmed through linear regression analysis. We found an interaction between symptom scores and group affiliation on peripherally circulating cytokine levels for four of the cytokines. This report demonstrates that opioids seem to interact with self‐reported mental health symptoms in a way that impacts levels of cytokines. We propose that opioids might be associated with a pro‐inflammatory dampening and that this should be taken into consideration when analysing cytokine levels.

## Graphical abstract

In patients with chronic HCV infection, mental distress was positively associated with circulating cytokines levels in non‐OMT patients, but showed the opposite pattern in patients receiving OMT. Interaction analyses confirmed that OMT modified the relationship between symptom burden and cytokines, particularly the pro‐inflammatory TNF, IL‐2, IL‐8 and IL‐9. These results suggest that opioids may dampen pro‐inflammatory immune responses linked to psychological distress and should be considered in future cytokine studies.

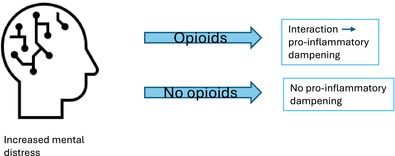

## 1 Introduction

Opioid maintenance treatment (OMT) has since its establishment proven its efficacy in terms of withdrawal symptom alleviation, reduced morbidity [1] and mortality [2] and reduction of crime rates [3]. For some time, it was believed that opioids were simply immunosuppressive, as demonstrated in both human [4] and animal [5, 6] studies investigating the immunological effect of morphine. Later studies emerged nuancing this perception, demonstrating that opioids could be both immunosuppressive and immunostimulatory [7, 8]. However, the relationship between opioids and cytokines does not seem to behave in a predictable, dichotomized manner. Cytokines themselves can be both pro‐ and anti‐inflammatory, exemplified by interleukin (IL)‐6 and transforming growth factor (TGF)‐β [9]. Moreover, reports on the immunomodulatory properties of methadone and buprenorphine, the most commonly used OMT drugs, show disparate results. For instance, whereas tumour necrosis factor (TNF) was higher in the OMT group after 1 year of treatment compared to baseline in one study [7], another study found no change in TNF after 6 months of treatment [8]. Similar divergent results were found for interferon (IFN)‐γ: It was higher at baseline than after more than 3 years of treatment in one study [10], but no change in levels was found after 6 months in another report [8]. This suggests that other factors than opioids also influence cytokine levels in OMT patients.

The reported incidence of mental health disorders among OMT patients varies from 32% [11] to 70% [12]. Also, between 43% [13] and 90% [14] of OMT patients have hepatitis C viral infection (HCV). Both HCV [15] and psychiatric symptoms [16] may influence cytokine levels. For example, increased mental health symptoms are often related to increased levels of proinflammatory cytokines [17], and interferon gamma‐induced protein (IP)‐10 (or C‐X‐C motif chemokine ligand 10, CXCL10) is higher in HCV‐infected patients compared to the noninfected OMT patients [18]. We have not found any studies investigating the joint effect of OMT and psychiatric symptoms on cytokine levels that also acknowledge the role of HCV. Relevant reports investigating cytokines and psychiatric symptoms among OMT patients either failed to report on infectious states [7, 19, 20] or excluded patients with clinically relevant liver disease [8]. This might be of importance, as increased levels of pro‐inflammatory cytokines associated with chronic inflammation influence neuroendocrine pathways and neurotransmitter systems [17]. Therefore, the joint effect might contribute to the pathophysiology of various mental health symptoms and disorders [17].

Our group has previously investigated associations between cytokine profile and psychiatric symptoms in a study sample from a general psychiatric in‐patient ward [21]. We found that only the group receiving psychotropic medications had a positive association between psychiatric symptoms and IP‐10 [21]. Other reports also demonstrated that when performing a group by factor analysis, associations previously not found appear: When dividing the study sample according to gender, the association between IL‐6 and depressive symptoms not found in the group as a whole was found only among females when performing a group by gender analysis [22]. Others have found that when linking different symptoms together, associations appear, suggesting that the symptoms interact and that it is the interaction that impacts the cytokine levels [23]. The aim of this study was therefore to investigate if there are any interactions between cytokines, self‐reported mental health symptoms and opioids by performing a group by opioid investigation. We hypothesized that there would be an enhancement of cytokine symptoms association among OMT patients.

## 2 Materials and Methods

This cross‐sectional study has been detailed in a previously published report [24]. Briefly, patients referred for anti‐HCV treatment were recruited from the Department of infectious diseases at the Nordland Hospital Trust, Bodø, Norway, in the period of April 2013 to December 2019. After informed consent, 120 patients out of 135 screened patients were included. Fifty‐three were OMT patients. The study was approved by the regional ethics committee (notification 2011/2024 REK and 2015/1808 REK) and was conducted in accordance with the Helsinki declaration.

Participants completed the Symptoms Check‐List‐90‐R (SCL‐90‐R) to assess psychological distress during the last week, each of which was rated on a 5‐point scale of distress [25]. The patients were interviewed with an adapted European Addiction Severity Index (Europ‐ASI) questionnaire [26] focusing on sociodemographics, physical health, economy, education and employment status, and alcohol and other drugs use. Body mass index (BMI) was calculated from the formula BMI = kg/m^2^. Current medication, smoking habits, allergies, previous medical history and ongoing withdrawal symptoms were recorded [24].

Venous blood was withdrawn and stored in Matrix tubes on ice up to 2 h before freezing at −80°C. The cytokine analysis was performed by multiplex technology with a predefined kit Bio‐Plex Human Cytokine 27‐Plex Panel (Bio‐Rad Laboratories Inc., Hercules, CA) according to the instructions of the manufacturer using three different batches [24].

Cytokines were log10‐transformed for normal distribution. Group comparisons used Mann–Whitney U (continuous/nonnormal) or chi‐square (categorical). Correlation analyses used Pearson's r correlation factor with *p*‐values < 0.05 as statistically significant. Multiple linear regression analysis was performed with cytokines as dependent variables and self‐reported symptom clusters and confounding factors (age, gender, BMI and smoking) as independent variables. Statistical analyses were performed using IBM SPSS Statistics viewer version 29.0.1.0 [24].

## 3 Results

Regarding demographic group characteristics, 53 participants were on OMT and 67 were not on OMT (Table 1). Only age was significantly different between the groups: Median age in the opioid group was 42 and in the nonopioid group 47 (*p* = 0.038). Other demographic variables, virus load and drug use during the last 30 days were not significantly different nor were mean self‐reported symptom cluster scores.

**TABLE 1:** Demographic differences between non‐OMT and OMT patients and virus load, self‐reported symptom cluster and substance use last 30 days.

		Non‐OMT	OMT	*p*
		*N* = 67 (56%)	*N* = 53 (44%)	
Demographics				
Female gender	N (%)	26 (39)	13 (25)	0.097
Age (years)	Median (IQR)	47 (37–56)	40 (34–51)	**0.038**
On psychotropic drugs	N (%)	15 (22)	19 (35)	0.236
BMI (kg/m^2^)	Median (IQR)	25 (22–28)	26 (23–30)	0.421
Smoking	N (%)	39 (58)	32 (61)	0.140
Norwegian nationality	N (%)	60 (90)	47 (89)	0.462
Virus load HCV RNA (IU/L*1000)	Median (IQR)	1255 (435–3297)	633 (276–3110)	0.154
Mental health scores (cluster) from SCL‐90‐R				
Depression	Mean (SD)	1.23 (0.85)	1.39 (0.90)	0.370
Anxiety	Mean (SD)	1.04 (0.84)	1.21 (0.79)	0.207
Somatization	Mean (SD)	1.30 (0.77)	1.21 (0.74)	0.588
Psychoticism	Mean (SD)	0.47 (0.55)	0.42 (0.36)	0.596
Phobic anxiety	Mean (SD)	0.82 (0.92)	0.80 (0.71)	0.569
Paranoid ideation	Mean (SD)	0.70 (0.71)	0.61 (0.59)	0.589
Obsessive–compulsive	Mean (SD)	1.38 (0.90)	1.45 (0.87)	0.628
Interpersonal sensibility	Mean (SD)	0.76 (0.68)	0.83 (0.70)	0.536
Hostility	Mean (SD)	0.62 (0.66)	0.49 (0.43)	0.510
Substance use last 30 days				
Alcohol	*N* (%)	35 (53)	20 (43)	0.272
Cannabis	*N* (%)	20 (33)	21 (44)	0.268
Amphetamine	*N* (%)	9 (17)	4 (9)	0.222
Benzodiazepines	*N* (%)	11 (26)	17 (39)	0.218
Opioids	*N* (%)	3 (8)	1 (2)	0.229
Heroin	*N* (%)	1 (3)	1 (2)	0.882

*Note:* Significant values (*p* < 0.05) are shown in bold.

Abbreviations: BMI, body mass index; HCV, hepatitis C virus; IQR, interquartile range; IU, international units; OMT, opioid maintenance therapy; SD, standard deviation.

In the stratified correlation analysis, we found an overall trend in which correlations, albeit not all significant, between cytokines and symptoms were positive (blue colour) in the non‐OMT group (Table 2) and negative (purple colour) in the OMT group (Table 3). The order of the cytokines in the Tables 2 and 3 is arranged by the largest differences between the groups placed first. Five cytokines (TNF, IL‐2, IL‐8, IL‐9 and IP‐10) stood out in terms of the higher amount of significant cytokine/symptoms correlations. In addition, they had the greatest correlation but in an opposite way in which the correlation in the non‐OMT group was positive and in the OMT group negative.

**TABLE 2:** Pearson's r correlation between the nine different dimensions of SCL‐90‐R and the log‐transformed values for serum cytokines in the patients who were not in opioid maintenance treatment (non‐OMT).

SCL‐90‐r subscales for non‐OMT patients										
	LLOD	Depression	Anxiety	Somatization	Psychoticism	Phobic anxiety	Paranoid ideation	Obsessive‐compulsive	Interpersonal sensitivity	Hostility
TNF	10.84	0.20	0.24	0.27*	0.15	0.28*	0.19	0.21	0.24*	0.21
IL‐2	0.84	0.17	0.29*	0.27*	0.20	0.30*	0.23	0.22	0.24*	0.15
IL‐8	1.00	0.23	0.23	0.16	0.15	0.15	0.13	0.14	0.17	0.02
IL‐9	1.48	0.06	0.20	0.15	0.08	0.30*	0.19	0.04	0.21	0.22
IP‐10	3.28	−0.06	0.03	−0.03	−0.06	0.13	−0.02	−0.04	0.09	−0.02
IL‐6	0.40	0.05	0.09	0.09	0.06	0.10	0.05	0.07	0.14	0.01
IL‐15	6.32	0.11	0.11	0.13	0.12	0.03	0.05	0.15	0.04	0.04
IL‐4	0.36	−0.02	0.04	−0.07	0.16	0.10	0.05	0.05	0.04	0.04
IL‐17	1.24	0.09	0.13	0.25*	0.10	0.18	0.18	0.09	0.13	0.22
Eotaxin	0.40	0.04	0.06	0.03	0.16	0.06	0.02	0.12	0.04	0.06
IL‐13	0.24	0.02	0.00	0.07	0.02	0.10	0.03	−0.03	0.04	0.05
MIP‐1α	0.16	0.10	0.15	0.17	0.19	0.20	0.18	0.13	0.18	0.22
IL‐1β	0.04	0.01	−0.06	0.08	−0.09	−0.07	−0.09	−0.01	−0.02	−0.06
IL‐5	3.16	0.06	−0.09	0.09	0.03	−0.12	0.03	0.05	−0.02	0.08
IL‐1Ra	18.40	−0.08	−0.03	0.02	0.16	0.16	0.03	−0.07	0.02	0.07
IFN‐γ	0.36	−0.10	−0.09	−0.10	−0.17	−0.16	−0.23	−0.07	−0.11	−0.28*
MCP 1	7.40	0.10	0.03	0.04	0.17	0.01	0.02	0.15	0.15	0.04
MIP‐1β	1.48	0.00	−0.07	−0.08	0.02	−0.17	−0.13	0.00	−0.08	−0.10
VEGF	12.44	0.06	−0.10	−0.12	−0.05	−0.20	−0.18	−0.05	−0.15	−0.14
IL‐12 p70	1.04	−0.10	−0.04	−0.04	−0.21	−0.07	−0.10	−0.17	−0.12	−0.06
PDGF BB	2.56	−0.02	0.06	0.14	0.11	0.07	0.06	0.13	0.08	0.04
RANTES	14.08	−0.04	−0.03	−0.08	−0.03	−0.13	−0.14	0.09	−0.06	−0.09

*Note:* The order of the cytokines is by the largest difference between non‐OMT and OMT patients being listed first. Values in blue colour indicate positive correlations and red/purple colour negative correlations. Denser colour with higher values.

Abbreviations: LLOD, lower limit of detection; OMT, opioid maintenance treatment.

* *p* < 0.05.

** *p* < 0.01.

**TABLE 3:** Pearson's *r* correlation between the nine different dimensions of SCL‐90‐R and the log‐transformed values for serum cytokines in the patients who were in opioid maintenance treatment (OMT).

SCL‐90‐r subscales for OMT patients										
	LLOD	Depression	Anxiety	Somatization	Psychoticism	Phobic anxiety	Paranoid ideation	Obsessive‐compulsive	Interpersonal sensitivity	Hostility
TNF	10.84	−0.28*	−0.33*	−0.20	−0.22	−0.29*	−0.28*	−0.27	−0.20	−0.29*
IL‐2	0.84	−0.30*	−0.21	−0.26	−0.24	−0.13	−0.37**	−0.35**	−0.21	−0.14
IL‐8	1.00	−0.15	−0.25	−0.05	−0.14	−0.21	−0.29*	−0.11	−0.10	−0.11
IL‐9	1.48	−0.21	−0.36**	−0.23	−0.28*	−0.14	−0.20	−0.36**	−0.23	−0.19
IP‐10	3.28	−0.30*	−0.31*	−0.13	−0.25	−0.32*	−0.34*	−0.36**	−0.30*	−0.22
IL‐6	0.40	−0.15	−0.15	−0.22	−0.02	−0.16	−0.10	−0.12	−0.01	−0.04
IL‐15	6.32	−0.09	0.05	−0.05	0.09	0.07	−0.06	0.02	0.06	0.23
IL‐4	0.36	−0.20	−0.04	−0.03	−0.02	0.02	−0.14	−0.18	−0.05	0.12
IL‐17	1.24	−0.08	−0.02	0.00	−0.09	0.01	−0.03	0.02	0.07	0.19
Eotaxin	0.40	−0.12	0.05	0.04	0.05	0.13	−0.05	0.17	0.03	−0.04
IL‐13	0.24	−0.14	−0.05	−0.02	−0.29*	0.01	−0.10	−0.23	−0.12	−0.18
MIP‐1α	0.16	−0.05	−0.05	0.17	−0.15	−0.13	−0.10	0.10	0.08	−0.02
IL‐1β	0.04	−0.11	−0.07	−0.12	−0.16	−0.06	−0.18	−0.11	−0.04	−0.12
IL‐5	3.16	0.04	0.04	0.06	0.11	−0.11	0.07	0.02	0.13	0.13
IL‐1Ra	18.40	−0.08	−0.01	0.00	−0.16	0.15	−0.04	−0.07	0.09	0.08
IFN‐γ	0.36	−0.10	−0.17	−0.05	0.02	−0.19	−0.13	0.02	−0.04	−0.18
MCP 1	7.40	0.12	0.09	0.10	0.17	0.03	0.16	0.18	0.18	0.25
MIP‐1β	1.48	0.05	0.09	0.08	0.04	−0.08	0.03	0.17	0.10	0.04
VEGF	12.44	0.13	0.13	0.16	0.16	0.04	0.08	0.24	0.18	0.10
IL‐12 p70	1.04	0.02	−0.01	0.07	−0.13	0.16	−0.10	−0.15	0.02	−0.07
PDGF BB	2.56	0.15	0.06	0.06	−0.01	0.28	0.09	0.07	0.21	0.05
RANTES	14.08	0.14	0.19	0.02	0.08	0.09	0.12	0.24	0.26	0.10

*Note:* The order of the cytokines is by the largest difference between non‐OMT and OMT patients being listed first. Values in blue colour indicate positive correlations and red/purple colour negative correlations. Denser colour with higher values.

Abbreviations: LLOD, lower limit of detection; OMT, opioid maintenance treatment.

* *p* < 0.05.

** *p* < 0.01.

In a group wise multiple linear regression analysis for these cytokines correcting for variables commonly known to confound the level of cytokines (gender, age, BMI and smoking), TNF showed a positive association with somatization (*β* = 0.40, *p* = 0.008), phobic anxiety (*β* = 0.30, *p* = 0.046), interpersonal sensitivity (*β* = 0.36, *p* = 0.018) and hostility (*β* = 0.35, *p* = 0.021) subscales of SCL‐90‐R among the non‐OMT patients (Table 4). In the OMT group, there was a negative association with depression (*β* = −0.37, *p* = 0.034), anxiety (*β* = −0.38, *p* = 0.031), somatization (*β* = −0.35, *p* = 0.047) and obsessive‐compulsive (*β* = −0.35, *p* = 0.048) subscales. For IL‐8, there was a positive relationship with depression (*β* = 0.30, *p* = 0.026), anxiety (*β* = 0.30, *p* = 0.042), interpersonal sensitivity (*β* = 0.29, *p* = 0.046) and hostility (*β* = 0.31, *p* = 0.035) subscales of the SCL‐90‐R among the non‐OMT patients, whereas for IP‐10, there was a negative relationship with depression (*β* = −0.49, *p* = 0.001), anxiety (*β* = −0.34, *p* = 0.037), phobic anxiety (*β* = −0.37, *p* = 0.018), paranoid ideation (*β* = −0.45, *p* = 0.004), obsessive‐compulsive (*β* = −0.41, *p* = 0.012) and interpersonal sensitivity (*β* = −0.41, *p* = 0.009) subscales of the SCL‐90‐R among the OMT patients. There were no significant associations in any of the groups for IL‐2 after adjustment for the confounders. For further details, see Table 4.

**TABLE 4:** Linear regression analysis of the five cytokines with the largest value difference between the non‐OMT and OMT patients in the symptom clusters of SCL‐90.

	TNF				IL‐2				IL‐8				IL‐9				IP‐10			
	Beta	95% CI	*p*	R square	Beta	95% CI	*p*	R square	Beta	95% CI	*p*	R square	Beta	95% CI	*p*	R square	Beta	95% CI	*p*	R square
Non‐OMT patients																				
Depression	0.27	−0.01; 0.19	0.061	0.17	0.12	−0.06; 0.16	0.377	0.18	0.30	0.01; 0.19	**0.026**	0.26	−0.01	−0.22; 0.21	0.939	0.19	−0.07	−0.14; 0.09	0.658	0.03
Anxiety	0.29	−0.00; 0.22	0.059	0.17	0.19	−0.05; 0.21	0.202	0.19	0.30	0.00; 0.20	**0.042**	0.25	0.14	−0.13; 0.35	0.358	0.20	0.06	−0.11; 0.15	0.735	0.03
Somatization	0.40	0.04; 0.27	**0.008**	0.23	0.24	−0.02; 0.24	0.098	0.21	0.27	−0.01; 0.21	0.069	0.23	0.12	−0.15; 0.36	0.402	0.20	−0.06	−0.16; 0.11	0.726	0.03
Psychoticism	0.18	−0.07; 0.25	0.255	0.13	0.14	−0.10; 0.25	0.366	0.18	0.20	−0.05; 0.24	0.187	0.20	−0.05	−0.38; 0.28	0.764	0.19	−0.05	−0.20; 0.15	0.781	0.03
Phobic anxiety	0.30	0.00; 0.18	**0.046**	0.18	0.21	−0.03; 0.17	0.153	0.20	0.19	−0.03; 0.14	0.194	0.20	0.29	0.01; 0.38	**0.040**	0.26	0.26	−0.02; 0.18	0.096	0.08
Paranoid ideation	0.29	−0.01; 0.23	0.063	0.17	0.20	−0.04; 0.22	0.184	0.20	0.25	−0.02; 0.20	0.095	0.22	0.09	−0.18; 0.34	0.538	0.19	−0.04	−0.15; 0.12	0.833	0.02
Obsessive‐compulsive	0.26	−0.01; 0.17	0.076	0.16	0.13	−0.06; 0.15	0.369	0.18	0.18	−0.03; 0.14	0.197	0.20	−0.02	−0.21; 0.19	0.915	0.19	−0.14	−0.15; 0.06	0.358	0.04
Interpersonal sensitivity	0.36	0.03; 0.28	**0.018**	0.21	0.21	−0.04; 0.25	0.158	0.20	0.29	0.00; 0.24	**0.046**	0.24	0.14	−0.14; 0.41	0.339	0.20	0.09	−0.11; 0.19	0.570	0.03
Hostility	0.35	0.02; 0.25	**0.021**	0.20	0.18	−0.05; 0.22	0.220	0.19	0.31	0.01; 0.22	**0.035**	0.25	0.14	−0.13; 0.37	0.346	0.20	−0.01	−0.14; 0.14	0.976	0.02
OMT patients																				
Depression	−0.37	−0.13; −0.01	**0.034**	0.32	−0.28	−0.19; 0.03	0.147	0.13	−0.29	−0.17; 0.02	0.120	0.17	−0.20	−0.21; 0.03	0.127	0.59	−0.49	−0.30; −0.08	**0.001**	0.54
Anxiety	−0.38	−0.15; −0.01	**0.031**	0.32	−0.05	−0.15; 0.11	0.789	0.06	−0.44	−0.23; −0.02	**0.018**	0.27	−0.26	−0.27; −0.00	**0.046**	0.61	−0.34	−0.29; −0.01	**0.037**	0.41
Somatization	−0.35	−0.18; −0.00	**0.047**	0.30	−0.12	−0.20; 0.11	0.551	0.07	0.05	−0.12; 0.16	0.810	0.09	−0.32	−0.36; −0.05	**0.012**	0.58	−0.08	−0.23; 0.14	0.653	0.31
Psychoticism	−0.19	−0.29; 0.10	0.302	0.22	−0.20	−0.49; 0.16	0.314	0.09	−0.15	−0.40; 0.18	0.446	0.11	−0.18	−0.60; 0.12	0.182	0.58	−0.29	−0.68; 0.05	0.085	0.38
Phobic anxiety	−0.34	−0.16; 0.00	0.056	0.30	−0.14	−0.17; 0.08	0.482	0.09	−0.27	−0.20; 0.03	0.148	0.17	−0.17	−0.25; 0.05	0.191	0.58	−0.37	−0.33; −0.03	**0.018**	0.46
Paranoid ideation	−0.30	−0.20; 0.02	0.090	0.27	−0.32	−0.33; 0.03	0.097	0.15	−0.24	−0.26; 0.06	0.208	0.14	−0.09	−0.28; 0.14	0.518	0.56	−0.45	−0.47; −0.10	**0.004**	0.49
Obsessive‐compulsive	−0.36	−0.14; −0.00	**0.048**	0.30	−0.30	−0.21; 0.03	0.127	0.14	−0.21	−0.17; 0.05	0.285	0.13	−0.24	−0.25; 0.02	0.079	0.60	−0.41	−0.30; −0.04	**0.012**	0.45
Interpersonal sensitivity	−0.25	−0.14; 0.03	0.163	0.24	−0.14	−0.19; 0.09	0.483	0.08	−0.23	−0.20; 0.05	0.215	0.14	−0.15	−0.25; 0.07	0.250	0.57	−0.41	−0.35; −0.06	**0.009**	0.47
Hostility	−0.33	−0.26; 0.02	0.092	0.27	−0.13	−0.31; 0.17	0.547	0.07	−0.25	−0.33; 0.09	0.235	0.14	−0.19	−0.43; 0.10	0.200	0.58	−0.34	−0.52; 0.01	0.056	0.40

*Note:* Linear regression analysis with log_10_ transformed cytokine values as dependent variables and symptom cluster of SCL as independent variables, adjusted for gender, age, BMI and smoking. Significant values (*p* < 0.05) are shown in bold.

Abbreviations: beta, beta coefficient; BMI, body mass index; CI, confidence interval; OMT, opioid maintenance treatment.

**FIGURE 1:**
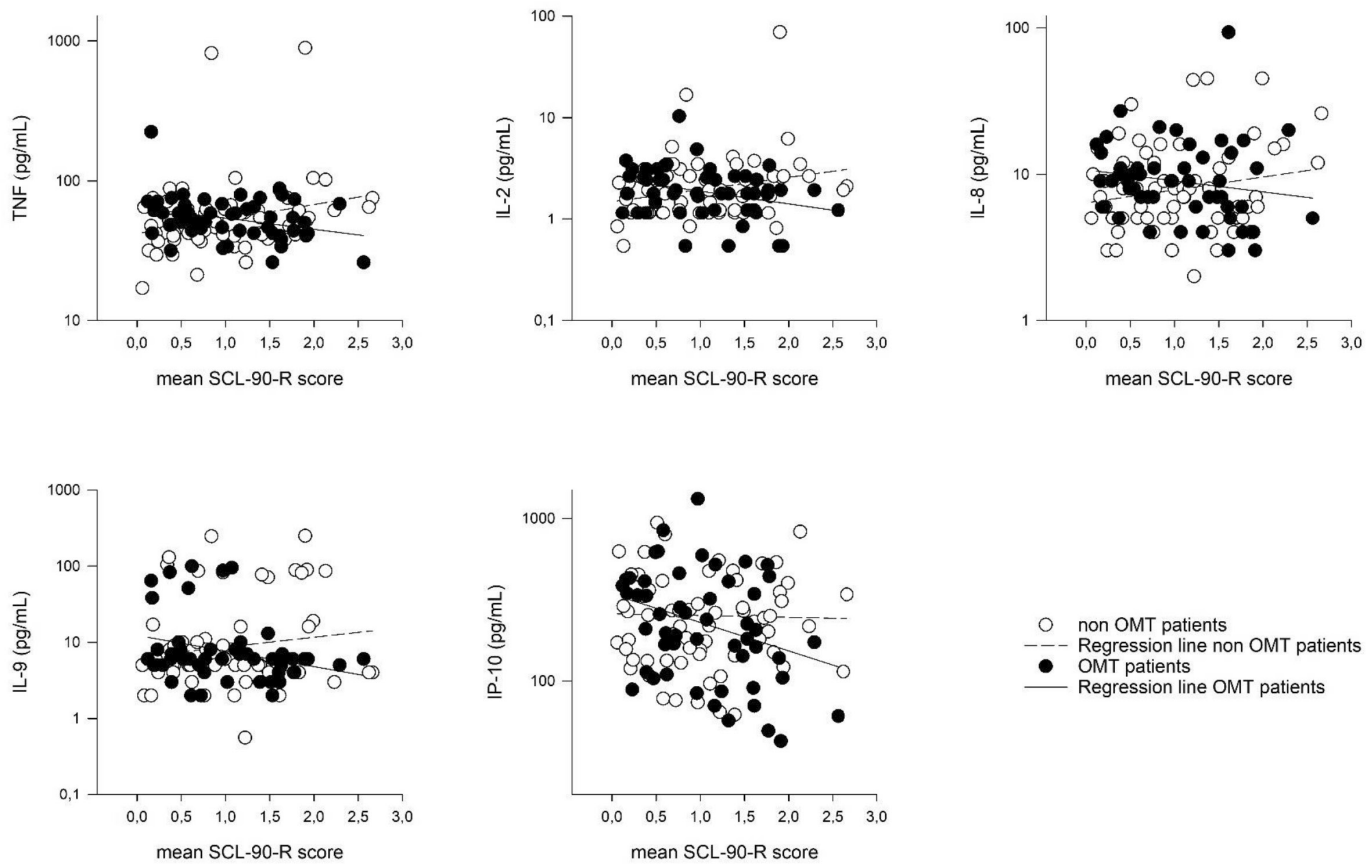
Mean of total SCL‐90‐R score for each of the patients on the x‐axis and cytokine levels on the y‐axis (log_10_ scale) with regression line of non‐OMT patients (dotted line) and OMT patients (full line).

For the five cytokines with the greatest difference in relation to the SCL‐90‐R subscales (TNF, IL‐2, IL‐8, IL‐9 and IP‐10), we plotted the cytokine serum levels against the mean SCL‐90‐R total score (Figure 1). Linear regressions with all the cytokines as dependent variables and the mean of all SCL‐90‐R symptom scores as independent variables for the two strata are shown in Table 5. In the non‐OMT group, there was a positive relationship between SCL‐90‐R and TNF and IL‐2 (*β* = 0.11, *p* = 0.028 and *β* = 0.12, *p* = 0.035, respectively). In the OMT group, there were negative relationships between SCL‐90‐R and all cytokines but IL‐8 (TNF: *β* = −0.08, *p* = 0.021; IL‐2: *β* = −0.13, *p* = 0.026; IL‐9: *β* = −0.21, *p* = 0.030; IP‐10: *β* = −0.18, *p* = 0.034, respectively). In order to test for the effect of any interaction between group affiliation and self‐reported symptom score on the cytokine levels, we ran a linear regression analysis of group affiliation × mean of all SCL‐90‐R scores as independent variable and the five cytokines as dependent variables. This showed an association with TNF (*p* = 0.004), IL‐2 (*p* = 0.003), IL‐8 (*p* = 0.047) and IL‐9 (*p* = 0.025) (Table 5). The association for IP‐10 was nonsignificant.

**TABLE 5:** Test for interaction through linear regression analysis between the five cytokines with the largest difference between non‐OMT and OMT patients and interaction term.

	Non‐OMT				OMT				Interaction term between group affiliation and mean of all symptom scores SCL‐90‐R
Cytokine	Beta	95% CI	*p*	R square	Beta	95% CI	*p*	R square	*p*
TNF	0.11	0.01; 0.20	**0.028**	0.07	−0.08	−0.15: −0.01	**0.021**	0.10	**0.004**
IL‐2	0.12	0.01; 0.23	**0.035**	0.26	−0.13	−0.23; −0.02	**0.026**	0.09	**0.003**
IL‐8	0.09	−0.02; 0.20	0.104	0.20	−0.08	−0.20; 0.05	0.217	0.17	**0.047**
IL‐9	0.13	−0.08; 0.34	0.225	0.02	−0.21	−0.39; −0.02	**0.030**	0.09	**0.025**
IP‐10	−0.01	−0.14; 0.11	0.854	0.02	−0.18	−0.34; −0.01	**0.034**	0.29	0.108

*Note:* Linear regression analysis with log_10_ transformed cytokine values as dependent variables and mean of all SCL‐90‐R scores as independent variables in both groups depicted in the first and second columns. Test for interaction between group affiliation and self‐reported symptom score on cytokine levels shown in the far‐right column showing linear regression analysis with log_10_ transformed cytokine levels as dependent variables and the interaction term (OMT group affiliation × mean of all SCL‐90‐R scores) as independent variable. Significant scores (*p* < 0.05) are shown in bold.

Abbreviations: beta, beta coefficient; CI, confidence interval; OMT, opioid maintenance treatment.

## 4 Discussion

In this cross‐sectional study sample of 120 HCV‐infected patients, we found an interaction between mental health symptoms and OMT group affiliation on the levels of cytokines. Whereas we observed an expected positive association between self‐reported mental health symptoms and pro‐inflammatory cytokines among the non‐OMT patients, we observed the opposite relationship with decreasing levels of peripherally circulating cytokines related to increasing mental health symptom score among the OMT patients. These findings were especially true for TNF, IL‐2, IL‐8 and IL‐9. These four cytokines are all pro‐inflammatory. The results were somewhat surprising since previous research has found a positive association between mental health symptom burden and pro‐inflammatory cytokines [17]. This may suggest a dampening of the pro‐inflammatory immune system in OMT patients [27].

Other ways to dampen the immune response have been demonstrated to occur through a bidirectional neurocircuit between the vagus nerve and the brain [28] and the bidirectional gut–brain axis [29]. Opioids exert their effect both in the central nervous system and in the gastrointestinal tract since opioid receptors are expressed in both areas [30]. In fact, opioids are a critical component in the gut–brain axis, and methadone causes alterations in the gut microbiome [30]. Whether our results may at least in part be explained by the OMT drugs' impact on these systems needs further elucidation.

Other reports have also demonstrated associations between mental health symptoms and cytokine in a group by factor manner. In a recent report, a hostility‐ and IP‐10 association was found in a group by psychotropic medication analysis, where only the patients on medication had a positive association [21]. This relates to our results, albeit with negative associations in the group receiving opioids. Others have found group by symptom intensity associations [23, 31, 32] and group by gender associations [32]. Collectively, our results seem not only to support the theory that variables interact in a multifaceted way causing interactions that impact cytokine levels [32]; we also corroborated this through our interaction‐term analysis demonstrating that opioids interact with self‐reported mental health symptom intensity in a way that impacts circulating pro‐inflammatory cytokines.

The opioids used in our study sample were buprenorphine and methadone. Buprenorphine is a partial agonist on the μ‐opioid receptor (MOR), a functional antagonist on the κ‐opioid receptor (KOR) and a weak antagonist on the δ‐opioid receptor (DOR) [33]. Methadone is a full agonist on the MOR, has some agonist action on the KOR and weak antagonist action on the DOR [33]. Studies have demonstrated that buprenorphine may have an antidepressant [34, 35] and antipsychotic [36] effect by its action on the KOR [37]. A significant antidepressant effect has also been found with methadone [38]. These findings and others demonstrating a protective neurocognitive effect of methadone [39] have led authors to suggest that opioids may have a neuroprotective effect by ways of various opioid receptor affinities [39, 40]. Our negative symptom/cytokine associations in the opioid‐group seem to confirm this hypothesis.

We investigated the mean of all the 90 self‐reported symptoms in the test for interaction. We found that abolishing the symptom cluster‐specific divisions yielded the same group difference with negative associations in the OMT group and opposite that of the non‐OMT group for four pro‐inflammatory cytokines. Other investigators have also found that a unidimensional approach accurately taps into the subscales of SCL‐90‐R [41]. This might imply that the impact on the cytokine levels comes from the total symptom burden itself rather than the nine‐factor symptom cluster specificity.

There are several methodological aspects to consider. Other studies have found the duration of OMT time to be correlated with cytokine levels and opioids in OMT patients [19]. Our cross‐sectional design offers no possibilities to study the change of cytokine levels as treatment progresses with time. Many data were missing for co‐administration of anti‐inflammatory [42] and psychotropic drugs [43] and smoking [44], all of which are known to influence cytokine levels. These might therefore have been insufficiently corrected for. The cytokines were analysed in three different kits, and interassay variability cannot be excluded [45]. Multiple analyses were conducted with no correction for multiple comparisons. These results need therefore to be seen as exploratory rather than definitive. The study lacks a healthy control group, eliminating the opportunity to compare and contextualize cytokine levels.

## 5 Conclusion

Opioids may influence psychological distress in ways associated with reduced pro‐inflammatory responses. We propose that opioids be taken into consideration for correctional purposes in future cytokine investigations.

## Author Contributions

All the authors meet the ICMJE authorship criteria and have made significant and equal contributions to this manuscript. All authors have read and agreed to the published version of the manuscript.

## Funding

The study was in part funded by research grant from Northern Norway Regional Health Authority (reference: RUS1303‐16). M.B. is supported by a NHMRC Leadership 3 Investigator grant (GNT2017131).

## Ethics Statement

The study was approved by the Regional ethics committee (notification 2015/1808/REK Nord and 2011/2024/REK Nord) and adhered to the principles of the Declaration of Helsinki. All participants in the study were given verbal and written detailed information about the study, and written consent was obtained from everybody.

## Conflicts of Interest

The authors have declared that no competing interests exist.

## Data Availability

Data will be made available upon direct contact with the corresponding author.
